# Emotion has no impact on attention in a change detection flicker task

**DOI:** 10.3389/fpsyg.2015.01592

**Published:** 2015-10-20

**Authors:** Robert C. A. Bendall, Catherine Thompson

**Affiliations:** Directorate of Psychology and Public Health, School of Health Sciences, University of SalfordSalford, UK

**Keywords:** emotion, visual attention, change detection, mood, broaden-and-build theory, IAPS

## Abstract

Past research provides conflicting findings regarding the influence of emotion on visual attention. Early studies suggested a broadening of attentional resources in relation to positive mood. However, more recent evidence indicates that positive emotions may not have a beneficial impact on attention, and that the relationship between emotion and attention may be mitigated by factors such as task demand or stimulus valence. The current study explored the effect of emotion on attention using the change detection flicker paradigm. Participants were induced into positive, neutral, and negative mood states and then completed a change detection task. A series of neutral scenes were presented and participants had to identify the location of a disappearing item in each scene. The change was made to the center or the periphery of each scene and it was predicted that peripheral changes would be detected quicker in the positive mood condition and slower in the negative mood condition, compared to the neutral condition. In contrast to previous findings emotion had no influence on attention and whilst central changes were detected faster than peripheral changes, change blindness was not affected by mood. The findings suggest that the relationship between emotion and visual attention is influenced by the characteristics of a task, and any beneficial impact of positive emotion may be related to processing style rather than a “broadening” of attentional resources.

## Introduction

The visual world is cluttered and it is impossible to attend to all items and areas simultaneously. Priority is therefore given to the most relevant areas or objects within a scene. This ‘biasing’ of attentional resources, known as selective visual attention, is subject to a range of influences and is dependent upon top–down and bottom–up processing. Top–down processing is characterized by goal-directed behavior, for example the allocation of attention to items matching target-defining features and the inhibition of distractors (Shiffrin and Schneider, 1977; Driver and Bayliss, 1989). In contrast, bottom–up processing is the automatic capture of attention by salient information in the environment, regardless of task demand (e.g., Itti and Koch, 2000). It should, however, be noted that that the top–down and bottom–up viewpoint cannot adequately explain all attentional processing (Awh et al., 2012). Attention has also been shown to be biased by statistical irregularities learned over time that cannot be explained by top–down or bottom–up processing (Zhao et al., 2013).

Traditionally, research in the field of visual attention has focused on the influences common to all individuals, for instance task demand (e.g., Henderson et al., 1999), past experience (e.g., Brockmole and Henderson, 2006), stimuli properties (e.g., Treisman and Gelade, 1980), and visual biases (e.g., Tatler, 2007). However, more recent work has explored influences upon visual attention that vary between individuals, such as individual differences and expertise (e.g., Sobel et al., 2007; Konstantopoulos et al., 2010). One such influence is the effect of emotion, and the number of studies investigating the impact of emotion on visual attention (and cognition in general) is growing rapidly.

A predominant model in this field is the broaden-and-build theory (Fredrickson, 1998, 2001). The theory proposes that positive emotions, including joy, interest, contentment, pride, and love, have the ability to “broaden” an individual’s “thought–action repertoires”. It is also suggested that positive emotions “build” an individual’s “enduring resources”, including physical resources (e.g., life longevity; Danner et al., 2001), intellectual resources (e.g., theory of mind; Leslie, 1987), social resources (e.g., relationship quality; Aron et al., 2000), and psychological resources (e.g., resilience; Fredrickson et al., 2003). Specifically, the theory posits that, over time, experiencing positive emotions will have a cumulative effect; enabling an individual to become more creative, knowledgeable, resilient, socially integrated, and healthy, and providing them with resources that can be utilized as necessary in the future. Frederickson outlines that negative emotions have the opposite effect; preventing one from thinking broadly and from building lasting psychological reserves, however, the theory is unique in that it focuses primarily on the positive influence of emotion. For instance, Fredrickson and Branigan (2005) propose that positive emotions have an evolutionary advantage over time as they build resources, whilst negative emotions have an evolutionary advantage ‘in the moment’ as they allow an individual to focus on a threat and the response to a threat.

Initial support for the broaden-and-build theory came from research suggesting that positive emotions result in an expansion of thoughts and actions (Fredrickson, 1998; Fredrickson and Branigan, 2001). Individuals experiencing positive mood states display increased levels of flexibility (Isen and Daubman, 1984), openness to information (Estrada et al., 1997), creativity and social openness (Isen et al., 1987; Baas et al., 2008; Garland et al., 2010) and enhanced semantic processing (Rowe et al., 2007). These studies demonstrate the ability of positive emotion to broaden cognition. However, they employ a range of tasks to measure the hypothesized expansion of thought–action repertoires and do not specifically measure the broadening of attention.

One task used to investigate the prediction that emotion affects the spread of attention is the global–local processing task (Navon, 1977, 2003). In this task participants are presented with large letters (global stimuli) composed of smaller letters (local stimuli) and are asked to respond to either the global or local feature. Findings consistently show a global precedence effect whereby responses are faster at the global level than the local level, and responses to local features are slowed when these are incompatible with the global feature (e.g., a global “H” comprised of local “S” compared to a global “H” comprised of local “H”). Research using this task has shown that negative moods promote a local processing style (with participants focusing more attention on the small letters) and positive moods induce a global processing style (with more attention directed to the large letter). It is argued that this reveals the narrowing of attention in negative moods and the broadening of attention in positive moods (e.g., Derryberry and Tucker, 1994; Basso et al., 1996). A study by Fredrickson and Branigan (2005) also utilized a global–local visual processing task, and found that positive mood resulted in larger global biases compared to neutral mood. Participants were asked to make a comparison judgment on two sets of stimuli comprising both local and global features. The stimuli could be compared at a global or a local level and findings revealed that participants in a positive mood were more likely to compare on the basis of global features. The study additionally showed that negative mood did not have a narrowing effect on processing (in contrast to earlier findings).

Fredrickson and Branigan (2005) conclude that positive mood states broaden attentional resources; however, the global precedence effect would indicate that global processing is the preferred processing style, and will be triggered automatically when a stimulus is presented. In addition, more resources are required to process information at a local level (in order to inhibit the global feature). This does not fit with the notion that the scope of attention expands due to positive emotions (or narrows due to negative emotions). We would therefore argue that positive affect is simply enhancing the bias toward global processing and not expanding the scope of attention. It is also suggested that the global–local processing task is not ideal for studying the benefits of positive emotion predicted in the broaden-and-build theory.

Whilst there are limitations in using a global–local processing task to investigate the effects of emotion on attention, other researchers provide support for the broaden-and-build theory using alternative methods. In an eye-tracking study, Wadlinger and Isaacowitz (2006) presented participants with three images (one located in the center of the screen and two situated in the periphery). Participants induced into a positive mood state made more fixations on the peripheral stimuli compared to those induced into a neutral mood state. They conclude that greater search in the periphery provides evidence for a broadening of visual attention due to positive emotion. However, the researchers compared eye movements in the periphery and they did not take into account any eye movements to the central image. The suggestion that positive moods broaden attention is specific in that individuals do not simply allocate attention more widely (potentially at the expense of processing information at the center), but that the resources available when one is in a positive mood are enhanced. This should allow for an expansion of attention whereby increased processing of peripheral information comes with no associated cost to processing central information. The study conducted by Wadlinger and Isaacowitz (2006) is thus unable to conclude that positive moods expand attentional resources and it is argued that the findings are more indicative of a bias toward viewing the displays at a more global level (participants pay attention to the peripheral images at the expense of focal information). In addition, their findings show that the increased eye movements to the peripheral images only occurred when these images were positive and therefore consistent with the mood state. Again, without monitoring eye movements to the central image it is impossible to conclude whether these findings support a broadening effect, or if they merely represent a bias of attentional resources toward mood-congruent information.

Rowe et al. (2007) also argue that they have evidence of positive emotions broadening attention. In their experiment, positive and negative affect was induced using music with differing mood altering properties (they also included a neutral condition in which participants were instructed to read information about Canada). In a modified version of the Eriksen flanker task (Eriksen and Eriksen, 1974) they presented participants with a central target that was flanked by compatible or incompatible distracters. The distance between the target and distracters was manipulated and findings showed that participants induced with positive mood suffered more interference from far distance distracters compared to those induced with neutral and sad moods (in the positive condition participants showed significantly slower response times to targets when they were incompatible to the distracters).

This evidence (albeit showing a negative impact of positive emotions) would initially suggest that in positive moods attention will expand, therefore allowing an individual to process more information (regardless of whether this information is relevant or irrelevant to the task). Yet other studies have failed to replicate these findings and there is now a growing body of literature that casts doubt on the broaden-and-build theory (and in particular, those findings demonstrating a broadening of visual attention under positive mood conditions). For example, a study by Bruyneel et al. (2013) partially replicated the flanker experiment of Rowe et al. (2007) but found no evidence for a broadening of attention on the basis of positive mood. In a second study Bruyneel et al. (2013) adopted a more ecologically valid mood induction procedure, whereby positive mood was induced by providing participants with positive feedback following completion of a task designed to induce stress (negative mood was induced by omitting this positive feedback and there was no neutral condition). An identical flanker task was adopted and results showed no interaction between mood, flanker compatibility, and flanker location on reaction times. The researchers did find that overall response times were larger in the negative mood condition (regardless of flanker location or compatibility), although they acknowledged this effect may have been due to a lack of counterbalancing in the experiment (the negative condition was always completed first therefore performance in the positive mood condition may have benefitted from a practice effect).

We propose that previous research investigating the effects of emotion on attention has been flawed due to the nature of the tasks utilized. Rather than explicitly measuring the scope of visual attention, many experimental paradigms have inadvertently measured processing strategy (and do not necessarily reveal the benefits of positive emotions predicted by the broaden-and-build theory). There are also many conflicting findings and results appear to vary according to task demand and stimuli valence. Further, whilst some studies do include conditions for positive, negative, and neutral mood states, other research fails to include either a negative condition, or a neutral (control) condition. As such, the precise way in which emotion affects attention cannot be concluded.

The current study sought to investigate whether emotion has an impact upon visual attention using a change detection flicker task. The change detection flicker paradigm (Rensink et al., 1997) involves presenting individuals with two images separated by a brief inter-stimulus interval (ISI). The images are identical except for one change and the task is to detect (and identify or locate) the change as quickly as possible. In the standard flicker paradigm, the images continue to alternate separated by the ISI until the change has been detected. Findings show that changes can take a long time to find (up to an average of 20 s; Shapiro, 2000), despite the change being found easily when the ISI is removed. The inability to find the change is termed ‘change blindness’ (Rensink et al., 1997) and the effect is explained through top–down and bottom–up allocation of attention. Under normal circumstances, when a change is made to the visual scene it elicits a motion transient that captures attention automatically. The ISI (or alternative, such as an eye movement) masks the motion transient and the change must therefore be found using a more controlled, effortful search.

The change detection flicker paradigm has been used to measure many of the influences upon visual attention and visual search. For example, changes made to salient features are detected faster than changes to less salient features, revealing an impact of bottom–up processing (e.g., Wright, 2005), and change blindness is affected by expertise (e.g., Werner and Thies, 2000), demonstrating the importance of top–down influences. Relevant to the current study, findings also show that under normal circumstances participants pay more attention to central and focal stimuli than to peripheral stimuli, resulting in improved change detection for central changes compared to peripheral changes (e.g., Rensink et al., 1997).

Change blindness is a robust effect and given the success of the change detection paradigm in measuring the allocation of attention, we propose that it is ideal for the current investigation. Participants will be presented with neutral images and asked to detect changes made to these images. Crucially the changes will be made to the center or to the periphery of each image. Three blocks of trials will be completed and prior to each one participant will be induced into positive, negative, or neutral mood states using images from the International Affective Picture System (IAPS; Lang et al., 2008). The IAPS is a large collection of images that have each been rated for valence and arousal and they have been successfully used to induce mood in a wide range of studies (Jiamsanguanwong and Umemuro, 2014; Lee et al., 2014; Limonero et al., 2015). On the basis of the broaden-and-build theory it is predicted that in the positive mood condition attentional resources will increase and peripheral changes will be detected significantly faster compared to the neutral condition. In contrast, peripheral change detection will be significantly slower in the negative condition. Critically, any benefit to peripheral change detection under positive mood should come with no associated cost to detection of central changes. This prediction is made on the assumption that the broaden-and-build theory argues for an overall expansion of attentional resources, rather than a bias to allocating resources to the wider surroundings at the expense of central information.

## Materials and Methods

### Participants

An opportunity sample of 51 (32 female) staff and students from the University of Salford aged between 18 and 44 years (*M* = 24.78, *SD* = 6.43) participated in this study. Written informed consent was gained from each participant after they were given procedural information regarding the experiment. Ethical approval was obtained from the College of Health and Social Care Ethics Panel at the University of Salford. All participants received a £10 inconvenience allowance.

### Design

A within-participants design was used with two independent variables; *mood* induced prior to the change detection task (positive, neutral, or negative), and *location* of the changing item in the change detection task (central or peripheral). The dependent variables consisted of accuracy (percentage correct) and response times (in seconds) to detect the changes. A measure of positive and negative affect was also recorded to validate the method for inducing emotion.

### Materials

The experiment was designed and run using E-Prime (Psychological Software Tools, Inc.) and participants completed the study using a Viglen Intel Quad Core computer with a 60 Hz, 19 inch monitor. Emotion was manipulated by presenting participants with visual images of differing emotional valence from the IAPS (Lang et al., 2008). A total of 80 images were selected, 20 positive (mean valence 7.65, mean arousal 5.05), 20 neutral (mean valence 4.62, mean arousal 3.11), and 20 negative (mean valence 2.35, mean arousal 5.17) images were presented during mood induction. Twenty additional positive images were presented at the end of the experiment (mean valence 7.87, mean arousal 5.13). No additional considerations were taken into account when choosing the experimental stimuli (i.e., the type of positive or negative mood evoked by each image). Please see Supplementary Material for a list of IAPS images used in this experiment. All images were presented in color and measured a maximum of 1024 × 768 pixels.

A total of 180 neutral images were used for the change detection task. Thirty six original images were taken by the authors (both indoor and outdoor scenes) and one central and one peripheral change was made to each image (making a further 72 images). All changes were deletions (one item in the scene disappeared) and care was taken to ensure that changes were all of a similar size. Central changes were made within the center of each image (within an area measuring 512 × 384 pixels) and peripheral changes were made outside of this area (see Supplementary Material). There were an equal number of peripheral changes made on the right and left side of the images. For each changed image a response screen was also created. This consisted of the original image separated into four equal sections that each contained a red letter (A, B, C, and D) to allow participants to indicate the location of a change. All images in the change detection task measured 1024 × 768 pixels.

The Positive and Negative Affect Schedule (PANAS; Watson et al., 1988), a 20-item self-report measure was used to record participants’ mood after presentation of affective stimuli. The measure consists of 20 words that describe positive and negative feelings and emotions. The words were presented in a random order and for each one participants were asked to “indicate to what extent you feel this way right now, that is, at the present moment” on a scale of 1 (very slightly or not at all) to 5 (extremely). The PANAS provides a measure of positive affect from the summed rating of all positive words and a measure of negative affect from the summed rating of the negative words. The minimum score for each measure is 10 (indicating low affect) and the maximum is 50.

### Procedure

**FIGURE 1:**
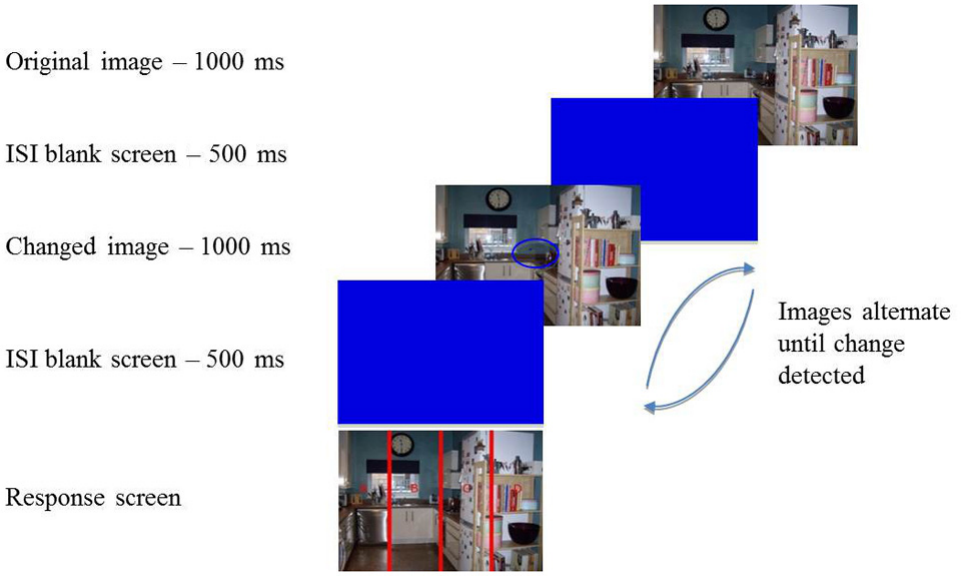
**Illustration of the change detection flicker task.** Participants were presented with an image for 1000 ms followed by an inter-stimulus interval (ISI; blank screen) for 500 ms. A changed image was then presented for 1000 ms followed by another ISI for 500 ms. This procedure continued until the participant identified the change in the scene and pressed the spacebar. They were then required to state where the change occurred by pressing the corresponding letter on the response screen. In this example the change was located in the center of the image and the correct response was ‘B’.

After providing written informed consent participants were seated approximately 22 inches from the screen and given full instructions about the task. If they were happy to proceed with the experiment they pressed the spacebar and were presented with 20 images from the IAPS. The images were shown for 5000 ms each in a random order, with a 500 ms ISI separating each one, and participants were asked to view these pictures naturally. Once all images had been presented on-screen instructions were given for participants to complete the PANAS. Following this participants pressed the spacebar again to begin the change detection task (**Figure 1**). A total of 24 change detection trials were completed, consisting of 12 central changes and 12 peripheral changes (6 to the left and 6 to the right). In each trial an image was presented for 1000 ms followed by a blue blank screen for 500 ms. The changed image was then presented for 1000 ms, again followed by the blue screen for 500 ms. Participants were instructed to search for the change between the two images and they continued to alternate until participants pressed the spacebar to indicate they had located the change. A response screen was then presented and participants reported the location of the change by pressing the relevant key on the keyboard (A, B, C, or D). Participants were told that the changes may be difficult to spot and if they were unable to locate the change they had the option of pressing ‘9’ to end a trial, however, they were asked to only use that as a “last resort”. All trials were presented in a random order.

Participants completed three blocks that each followed the above procedure (viewing of the IAPS images, completion of the PANAS, the change detection task). In the first block the IAPS images were either positive or negative (counterbalanced across participants). In the second block the images were always neutral, and in the final block they were again positive or negative depending on the valence of the first set of images. In the change detection task all 36 pairs of stimuli were shown twice (once with a central change and once with a peripheral change). The same image could not be presented twice in a single block.

Following completion of the final change detection task participants were presented with 20 further positive images from the IAPS (presented for 5000 ms each with an ISI of 500 ms). This was to make certain that participants were induced into a positive mood when leaving the lab. At this point they were also thanked for their time and given an inconvenience allowance.

## Results

Data collected included accuracy and response times to the change detection task and a positive and negative affect score from the PANAS. The analysis for each measure is presented below.

### Affect Scores

**FIGURE 2:**
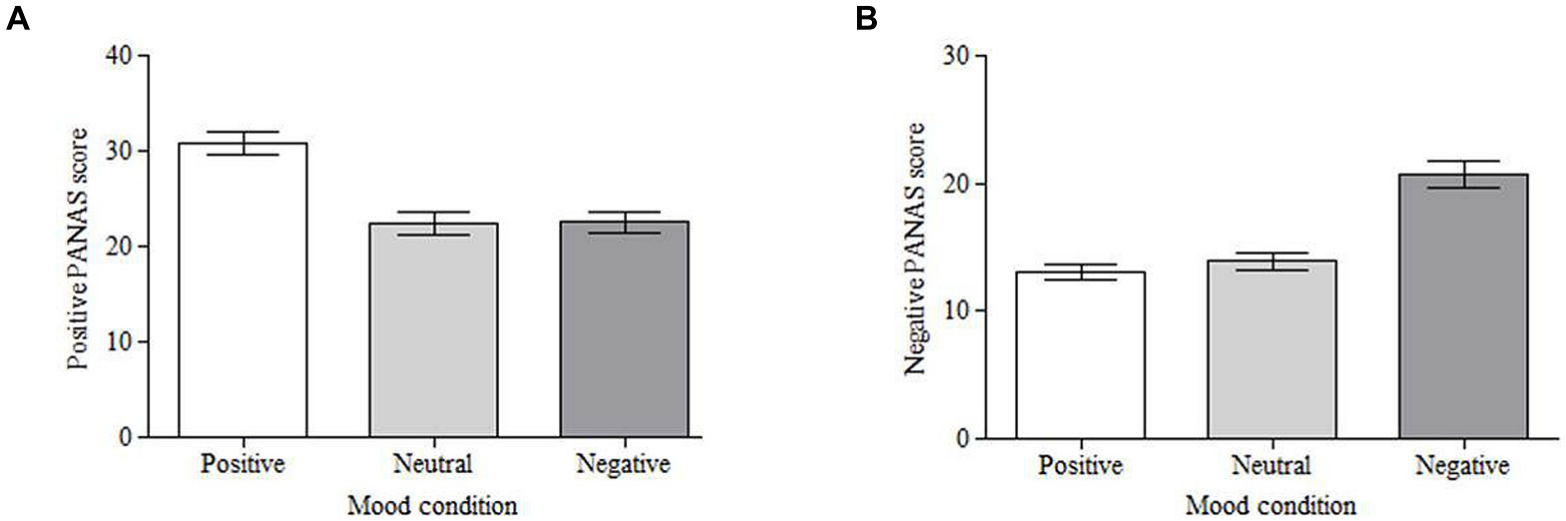
**Mean Positive and Negative Affect Schedule (PANAS) scores for each induced mood condition.** Self-reported mood varied across the conditions for both positive affect **(A)** and negative affect **(B)**. Error bars = standard error of the mean.

The affect scores were taken to validate the mood induction technique (presenting participants with images from the IAPS). Positive and negative scores were analyzed separately using a one-way repeated measures analysis of variance (ANOVA) followed by planned comparisons to compare the positive and negative conditions to the neutral condition. Analysis of the positive affect scores showed a significant effect of mood, *F*(2,100) = 36.12, *MSE* = 32.85, *p* < 0.001, partial η^2^ = 0.42. Planned comparisons show that viewing positive stimuli significantly increased positive mood scores compared to viewing neutral stimuli (*M* = 30.80, *SD* = 8.16 vs. *M* = 22.35, *SD* = 8.66; *F*(1,50) = 63.98, *MSE* = 56.93, *p* < 0.001, partial η^2^ = 0.56; **Figure 2A**). There were no differences in positive affect after viewing negative stimuli (*M* = 22.55) compared to neutral stimuli [*F*(1,50) = 0.031, *MSE* = 63.76, *p* = 0.862, partial η^2^ = 0.00; **Figure 2A**]. Analysis of negative affect also showed a significant effect of mood, *F*(2,100) = 45.97, *MSE* = 19.78, *p* < 0.001, partial η^2^ = 0.48. Planned comparisons reveal that viewing negative stimuli significantly increased negative mood scores compared to viewing neutral stimuli (*M* = 20.75, *SD* = 7.60 vs. *M* = 13.90, *SD* = 4.98; *F*(1,50) = 57.01, *MSE* = 41.90, *p* < 0.001, partial η^2^ = 0.53; **Figure 2B**). There was no difference in negative affect between the positive (*M* = 13.04) and neutral conditions [*F*(1,50) = 1.381, *MSE* = 27.48, *p* = 0.245, partial η^2^ = 0.03; **Figure 2B**].

### Change Detection Task

**FIGURE 3:**
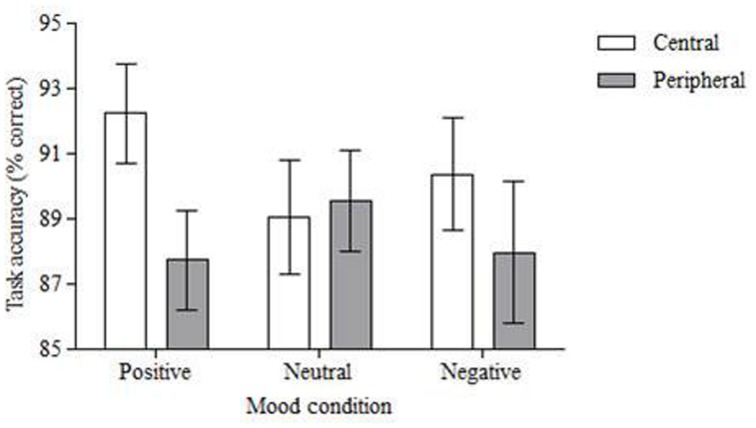
**Mean percentage accuracy in the change detection task.** Central changes were detected more accurately than peripheral changes but this did not vary according to the mood induced prior to the task. Error bars = standard error of the mean.

Two 2 (*location*) × 3 (*mood*) repeated measures ANOVAs were completed to investigate the influence of emotion on the allocation of attention in the change detection task. Analysis of accuracy revealed a significant effect of location, *F*(1,50) = 4.756, *MSE* = 0.01, *p* < 0.05, partial η^2^ = 0.09. Central changes were detected more accurately (*M* = 90.56%, *SD* = 11.91) than peripheral changes (*M* = 88.42%, *SD* = 12.59; see **Figure 3**). However, there was a non-significant effect of emotion, *F*(2,100) = 0.241, *MSE* = 0.02, *p* = 0.786, partial η^2^ = 0.01, and no significant interaction between location and emotion, *F*(2,100) = 2.127, *MSE* = 0.01, *p* = 0.125, partial η^2^ = 0.04.

**FIGURE 4:**
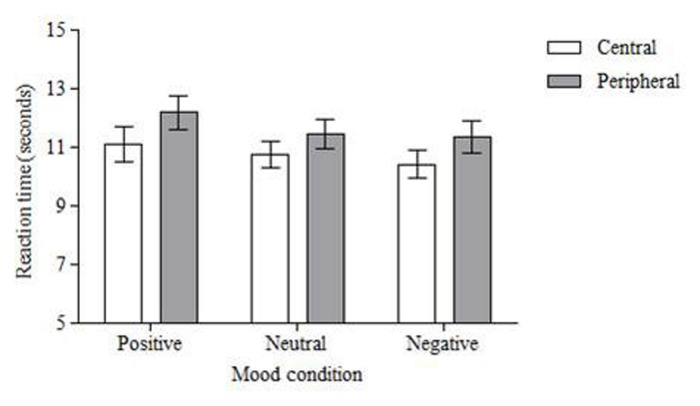
**Mean response time (in seconds) to correctly detect changes.** Central changes were detected faster than peripheral changes across all mood conditions. Error bars = standard error of the mean.

For reaction time there was again a significant effect of location, *F*(1,50) = 6.888, *MSE* = 9.005, *p* < 0.01, partial η^2^ = 0.12. Central changes were identified quicker than peripheral changes (*M* = 10.77 s, *SD* = 3.69 vs. *M* = 11.67 s, *SD* = 3.93; see **Figure 4**). There was no main effect of emotion, *F*(2,100) = 1.275, *MSE* = 12.395, *p* = 0.284, partial η^2^ = 0.03, and there was no interaction between location and emotion, *F*(2,100) = 0.075, *MSE* = 11.191, *p* = 0.928, partial η^2^ = 0.00.

## Discussion

The aim of the current study was to investigate the impact of emotion on visual attention. In particular the experiment was designed to measure whether positive emotions broaden visual attention, and whether negative emotions lead to attentional narrowing. This was explored using a change detection flicker task which allowed for the manipulation of ‘location’ whereby changes (centrally located or in the periphery) were made to images and participants had to detect these changes as quickly as possible. In accordance with the broaden-and-build-theory (Fredrickson, 1998, 2001), it was predicted that change blindness would reduce for changes in the periphery when participants were induced into positive mood but would increase when negative mood was induced (compared to a neutral condition).

The change detection flicker paradigm is a well-utilized method for studying attention, and differences between detection of central and peripheral changes have been evidenced in a number of studies. For instance, Rensink et al. (1997) found that changes to areas of central interest were detected faster than changes to peripheral areas. The current findings support this past research. Participants were significantly more accurate and significantly quicker to locate central changes than peripheral changes. This main effect is very important in endorsing the design of the flicker experiment. To establish any impact of emotion on the allocation of visual attention to central and peripheral locations it was vital to first show that visual attention and search followed the expected pattern, with resources located to the center of a scene before the periphery. We can therefore have confidence in the experimental paradigm.

Despite showing that attention is allocated to information at the center of a scene before being allocated to the periphery, there was no evidence that this varied according to mood. In contrast to past research (e.g., Fredrickson and Branigan, 2005; Wadlinger and Isaacowitz, 2006; Rowe et al., 2007) and contradicting the-broaden-and-build theory (Fredrickson, 1998, 2001), participants did not show a wider spread of attention when induced with positive mood. The current study therefore gives no support to the suggestion that positive emotions broaden attention (and that negative emotions lead to attentional narrowing). This finding was unexpected given that previous evidence has demonstrated an effect of emotion on attention (Fredrickson and Branigan, 2005; Wadlinger and Isaacowitz, 2006; Rowe et al., 2007). It should be noted that despite the non-significant interaction between mood and location in the change detection task, there was increased accuracy in detecting central changes compared to peripheral changes in the positive condition. However, even this non-significant trend is inconsistent with the predictions of the broaden-and-build theory as it provides no evidence for a broadening of spatial attention under positive mood conditions. At most we would argue that this effect may be partially responsible for the significant difference between detection accuracy of central and peripheral changes. In addition, in many change blindness studies (including the current experiment) the most important measure of performance is reaction time (rather than accuracy) and analysis of this dependent variable shows no comparable trend.

One possible explanation for the non-significant effect of emotion in the current study could be that participants were not successfully induced into the experimental mood conditions. For instance the PANAS self-report data were collected immediately after mood induction, and whilst analysis showed that emotion was successfully induced, it is not known whether this induced affect persisted throughout the change detection task. Given that the IAPS has been successfully used previously to induce emotion in research studies (Jiamsanguanwong and Umemuro, 2014; Lee et al., 2014; Limonero et al., 2015), we would argue that emotion was successfully induced but that this had no influence upon attention, however, in future it would be prudent to collect self-report data after each change detection block.

An alternative explanation for the current findings is that the experimental paradigm measures visual processing in a different way to some of the former studies. By manipulating the location of the changes the present study was able to investigate how visual attention is allocated across a real-world scene. Under standard viewing conditions an observer will allocate resources to the center of a display before attending to the periphery (e.g., Brockmole and Henderson, 2006; Tatler, 2007) therefore central changes will be detected faster than peripheral changes (as was found in the current experiment). If positive emotions expand the available attentional resources then the scope of attention should broaden (Fredrickson, 1998, 2001), allowing for faster (and more accurate) detection of peripheral changes compared to neutral or negative emotions (a prediction not supported by the current findings). In earlier studies this central/peripheral distinction was not possible. For instance, in some studies supporting the broaden-and-build theory participants are asked to make a judgment about one feature of a single stimulus or a small set of highly similar stimuli (the global–local task, e.g., Derryberry and Tucker, 1994; Basso et al., 1996, and the flanker task; e.g., Rowe et al., 2007). We argue that these tasks measure visual processing style (i.e., global or local) rather than the spatial allocation of attention and are therefore unable to test the claim that positive emotions expand attentional resources. Taking the past findings into consideration, the current results would indicate that positive moods do not enhance attentional resources; they merely bias the observer toward a particular method of processing information. This bias cannot be tested using the current methodology, yet incorporating global and local changes into a change detection paradigm may be one way to explore this further.

One study that comes close to measuring how visual attention is allocated through space and how emotion influences this was conducted by Wadlinger and Isaacowitz (2006). Similar to the current experiment they used natural scenes and measured search (via eye-tracking) to peripheral information. Their findings are, however, limited given that they did not analyze eye-movements to the center of the display, and they also utilized a display containing three separate images. This again does not allow for a true measure of how attention moves within a scene and lacks ecological validity. The study did support the broaden-and-build theory by showing that participants made more fixations on peripheral information under positive mood conditions, yet this only occurred when the information was mood-congruent. Supporting the findings of Wadlinger and Isaacowitz (2006), other studies have shown that the influence of emotion on cognition can be dependent upon the characteristics of the specific task stimuli used. For example, Grol et al. (2014) argue that positive emotion broadens attention but only when the stimuli are self-related. These findings suggest caution when interpreting the present results. It is possible that participants were induced into mood states as a result of viewing emotionally valenced stimuli (validated by the PANAS scores), only for the change detection task stimuli (which consisted of neutral images) to return mood to neutral.

The variation in findings in this field thus outline the importance of the experimental task used to investigate any impact of emotion on attention. The paradigms used range from very simple tasks with a relatively low level of difficulty (e.g., the flanker task) to more demanding tasks incorporating real-world stimuli. It is also possible that emotion has differing influences on overt attention (e.g., measured by Wadlinger and Isaacowitz (2006) and the current study) and covert attention (e.g., measured by Bradley et al., 2000; Rowe et al., 2007). This makes comparison across different experiments very difficult. It also suggests that any impact of emotion may be influenced by the characteristics of a task, for example the stimuli used and the demand of the task. This argument could be made about the paradigm used in the current study and it may be possible that the change detection flicker task was too difficult to elicit any influence of emotion. Whilst participants in the current experiment took significantly less time to detect changes (a mean of 11.3 s) compared to some change detection tasks (up to 20 s; Shapiro, 2000), the difficulty of the task may have masked any potential influence of emotion on change blindness. Completion of a change detection task requires cognitive control to allow for focused attention toward relevant information and inhibition of irrelevant information. Regulating emotion also involves cognitive control processes whereby an individual may try to inhibit an inappropriate or unwanted response. It is therefore highly likely that task difficulty interacts with any impact of emotion, a suggestion supported by the study of Jasinska et al. (2012). In their study using the multi-source interference task (Bush and Shin, 2006) participants were presented with three numbers and had to identify an oddball number with a corresponding button press. The spatial position of the oddball could be congruent or incongruent to the correct response and on some trials threatening or rewarding distracters were also presented. Responses were slower with both threatening and rewarding distracters (compared to no distractor) for incongruent trials, but not for congruent trials. These data demonstrate that task difficulty can mitigate the influence of emotion on attention.

A further influence that may have contributed to the impact of task difficulty was if participants were trying to regulate their emotions during the change detection task. Emotional distraction occurring within a cognitive task depends on interactions between cognitive systems that allow an individual to stay focused on the task, and those systems that are responsible for the processing of emotional information (Dolcos et al., 2011). Here it is proposed that the two systems compete for processing resources where emotional distractors result in bottom–up processing of task irrelevant information and adversely influence task performance. This deficit in task performance can be mitigated by utilizing top–down cognitive control processes. Detrimental influences of emotional distraction on task performance have been seen in studies using clinical and healthy populations (Jasinska et al., 2012; Krause-Utz et al., 2012). This is a possible avenue for further study as the current experiment did not take into account any influence of emotional regulation. However, if participants were using cognitive resources to manage their emotions and inhibit the emotional distraction following viewing of the IAPS stimuli we would expect better performance in the neutral condition compared to the both the positive and negative condition and this was not found. As a consequence it is unlikely that emotional regulation can explain the lack of any influence of emotion on attention in this study and instead other factors may play a role.

Cognitive theories attempt to explain behavior in terms of average group level performance; however, these models often fit less accurately when they are applied to individuals (Parasuraman and Giambra, 1991). It is therefore possible that individual differences can mitigate any effect of emotion on cognition. For example, state and trait negative affect have been shown to have separate and combined influences on attentional processing (Crocker et al., 2012). The complexity of the relationship between emotion and visual attention due to individual differences is demonstrated in a study conducted by Grol and De Raedt (2014). Facial stimuli of varying emotions (happy, sad, and neutral) were used to investigate the influence of stimuli valence (under positive and neutral mood) on attentional breadth and participants were given the task of locating a small target appearing at varying distances from the face. The researchers also took a measure of depressive symptoms using the Beck Depression Inventory (BDI-II; Beck et al., 1996). Mood and stimuli valence had no influence on attentional breadth and only an effect of distance was observed whereby accuracy was higher when the target was presented closer to the facial stimuli. However, participants with high BDI-II scores showed greater attentional narrowing in the task under positive emotion. Further, among individuals with high BDI-II scores, increases in positive mood were related to more pronounced attentional narrowing for positive stimuli. For participants with low BDI-II scores, increases in positive mood were related to attentional broadening for positive stimuli. It therefore appears that individual differences can mitigate the influence of emotion on visual attention. The current study did not take account of individual mood or depressive symptoms and this needs to be investigated further. Research investigating the role of individual differences on emotion and cognition is important as it will help to understand functioning in healthy populations as well as those factors that may increase susceptibility to a range of affective disorders.

## Conclusion

Using a change detection flicker task there was no evidence that mood affects visual attention. The findings are consistent with the view that research showing a broadening effect of positive emotion and a narrowing effect of negative emotion may instead demonstrate a bias toward a particular processing strategy rather than a broadening of the available attentional resources. Consequently, the broadening role of positive emotion on visual attention may not be as simple as the broaden-and-build theory (Fredrickson, 1998, 2001) outlines. We propose that studies using simplistic tasks in which participants attend to a very limited set of features cannot adequately test the effects of emotion on spatial attention. The change detection flicker task offers a more effective measure of attention under differing emotional conditions as (1) the spatial location of stimuli can be controlled to allow for a comparison of central and peripheral attention, (2) the experiment paradigm allows for the use of real-world stimuli, and (3) the task is more complex and this provides a method for exploring how individual differences in attentional control and mood moderate the influence of emotion on cognition. There is a growing body of research showing that any influence of emotion on visual attention is affected by task constraints (e.g., the type of stimuli and the valence of this stimuli) and task difficulty and these can all be manipulated using the change detection paradigm. Research also shows that individual differences mitigate the effects of emotion on attention and there is scope to expand on this work to investigate whether such influences have contributed to the current findings.

## Conflict of Interest Statement

The authors declare that the research was conducted in the absence of any commercial or financial relationships that could be construed as a potential conflict of interest.
